# TFEB Overexpression, Not mTOR Inhibition, Ameliorates RagC^S75Y^ Cardiomyopathy

**DOI:** 10.3390/ijms22115494

**Published:** 2021-05-23

**Authors:** Maengjo Kim, Linghui Lu, Alexey V. Dvornikov, Xiao Ma, Yonghe Ding, Ping Zhu, Timothy M. Olson, Xueying Lin, Xiaolei Xu

**Affiliations:** 1 Department of Biochemistry and Molecular Biology, Mayo Clinic, Rochester, MN 55901, USA; Kim.maengjo@mayo.edu (M.K.); lu-linghui@hotmail.com (L.L.); advornikov@email.arizona.edu (A.V.D.); Ma.xiao@mayo.edu (X.M.); Ding.yonghe@mayo.edu (Y.D.); Zhu.ping@mayo.edu (P.Z.); Lin.xueying@mayo.edu (X.L.); 2 Department of Cardiovascular Medicine, Mayo Clinic, Rochester, MN 55901, USA; Olson.timothy@mayo.edu; 3 School of Traditional Chinese Medicine, Beijing University of Chinese Medicine, Beijing 100029, China; 4 Department of Cellular and Molecular Medicine, The University of Arizona, Tucson, AZ 85721, USA; 5 Department of Pediatric and Adolescent Medicine, Division of Pediatric Cardiology, Mayo Clinic, Rochester, MN 55901, USA

**Keywords:** RagC^S75Y^, cardiomyopathy, Rags, mTOR, TFEB

## Abstract

A de novo missense variant in Rag GTPase protein C (RagC^S75Y^) was recently identified in a syndromic dilated cardiomyopathy (DCM) patient. However, its pathogenicity and the related therapeutic strategy remain unclear. We generated a zebrafish Rragc^S56Y^ (corresponding to human RagC^S75Y^) knock-in (KI) line via TALEN technology. The KI fish manifested cardiomyopathy-like phenotypes and poor survival. Overexpression of RagC^S75Y^ via adenovirus infection also led to increased cell size and fetal gene reprogramming in neonatal rat ventricle cardiomyocytes (NRVCMs), indicating a conserved mechanism. Further characterization identified aberrant mammalian target of rapamycin complex 1 (mTORC1) and transcription factor EB (TFEB) signaling, as well as metabolic abnormalities including dysregulated autophagy. However, mTOR inhibition failed to ameliorate cardiac phenotypes in the RagC^S75Y^ cardiomyopathy models, concomitant with a failure to promote TFEB nuclear translocation. This observation was at least partially explained by increased and mTOR-independent physical interaction between RagC^S75Y^ and TFEB in the cytosol. Importantly, TFEB overexpression resulted in more nuclear TFEB and rescued cardiomyopathy phenotypes. These findings suggest that S75Y is a pathogenic gain-of-function mutation in RagC that leads to cardiomyopathy. A primary pathological step of RagC^S75Y^ cardiomyopathy is defective mTOR–TFEB signaling, which can be corrected by TFEB overexpression, but not mTOR inhibition.

## 1. Introduction

Dilated cardiomyopathy (DCM), a progressive cardiac disorder that frequently leads to heart failure, is the most common indication for heart transplantation [1,2]. Mutations in more than 60 genes have been linked to DCM [2]; however, the genetic etiologies in ~60% of DCM patients remain elusive [3]. Through whole exome sequencing of a pediatric DCM patient, we recently identified a *de novo* S75Y variant in Rag GTPase protein C (RagC, or RRAGC), revealing *RRAGC* as a new susceptibility gene [4]. However, further evidence is required to prove pathogenicity, including functional studies in animal models and identification of additional RRAGC mutations in DCM cohorts.

RagC is a member of the Ras small GTPase superfamily, which forms heterodimeric Rags with other family members, like RagA–C or RagB–C [5]. The activity of Rags is dynamically switched by changing GDP/GTP binding status [6]. The Rags are active when RagA/B is bound to GTP and RagC/D to GDP (^GTP^RagA–RagC^GDP^), while the opposite nucleotide configuration (^GDP^RagA–RagC^GTP^) inactivates Rags [5]. The Rags are localized at the surface of lysosomes to regulate cellular metabolism in response to amino acid availability [7,8]. When nutrient is abundant, Rags are activated and bind to the Raptor subunit of mammalian target of rapamycin complex 1 (mTORC1) to recruit mTORC1 to the lysosome [9,10]. Activated Rags also recruits transcription factor EB (TFEB), a master transcriptional regulator of autophagy-lysosome machinery, to the lysosomal surface via direct interaction, where mTORC1 phosphorylates TFEB thus retaining TFEB in the cytosol [11]. Upon fasting or lysosome stress, Rags is inactivated, TFEB is dephosphorylated and translocated into nuclei to activate downstream lysosomal genes [11,12,13].

The S75 site in RagC is evolutionarily conserved from yeast to human beings, and is located in the phosphate-binding loop (P-loop) motif that is known to mediate GTP/GDP binding [14,15]. Many cases of RRAGC S75 mutation, including S75C, S75N, S75F, were identified from follicular lymphoma patients. These mutations resulted in greater GDP binding affinity and hyperactive mTORC1 signaling [15,16], suggesting a pivotal function of RRAGC S75 in activating Rags. Based on *in silico* protein modeling via computational structural analysis, it is predicted that the S75Y mutation increases the GDP-bound state [4], leading to the hypothesis that S75Y is also a gain-of-function (GOF) mutation. In line with this hypothesis, our previous studies found activation of the Rags–mTORC1 signaling pathway in 293 cells [4], prompting the current study using animal models and cardiomyocytes to further decipher molecular changes and to develop related therapeutic strategies.

There is extensive evidence for both mTOR and TFEB as therapeutic targets for cardiomyopathies of different etiology. First, hyperactive mTORC1 signaling is detrimental and mTOR inhibition exerts cardioprotective effects in a broad spectrum of heart diseases including pressure overload heart failure, inherited and metabolic cardiomyopathies [17,18]. Second, TFEB activation has been found to rescue cardiomyopathic manifestations in *Myh*-CryAB^R120G^ mice [19] and shows beneficial effects in myocardial ischemia-reperfusion injury [20]. Of note, the therapeutic benefit of TFEB is linked to autophagy regulation while genetic defects in autophagy have been shown to increase the propensity of laboratory animals to develop cardiovascular diseases [21]. Whether mTOR, TFEB, or both are effective therapeutic targets for RagC^S75Y^ cardiomyopathy remains untested.

In this study, we first set out to prove pathogenicity of the S75Y variant through establishing the first knock-in (KI) cardiomyopathy model in zebrafish. We then deciphered aberrant signaling events incurred by the S75Y variant in neonatal rat ventricle cardiomyocytes (NRVCMs), and revealed mTORC1 activation and TFEB inhibition. Surprisingly, mTOR inhibition failed to exert therapeutic effects. In contrast, TFEB activation was sufficient to ameliorate RagC^S75Y^ cardiomyopathy both *in vitro* and *in vivo*, suggesting a therapeutic strategy for RagC^S75Y^ cardiomyopathy.

## 2. Results

### 2.1. Rragc^S56Y^ KI Zebrafish Manifest Cardiomyopathy-Like Phenotypes

**Figure 1:**
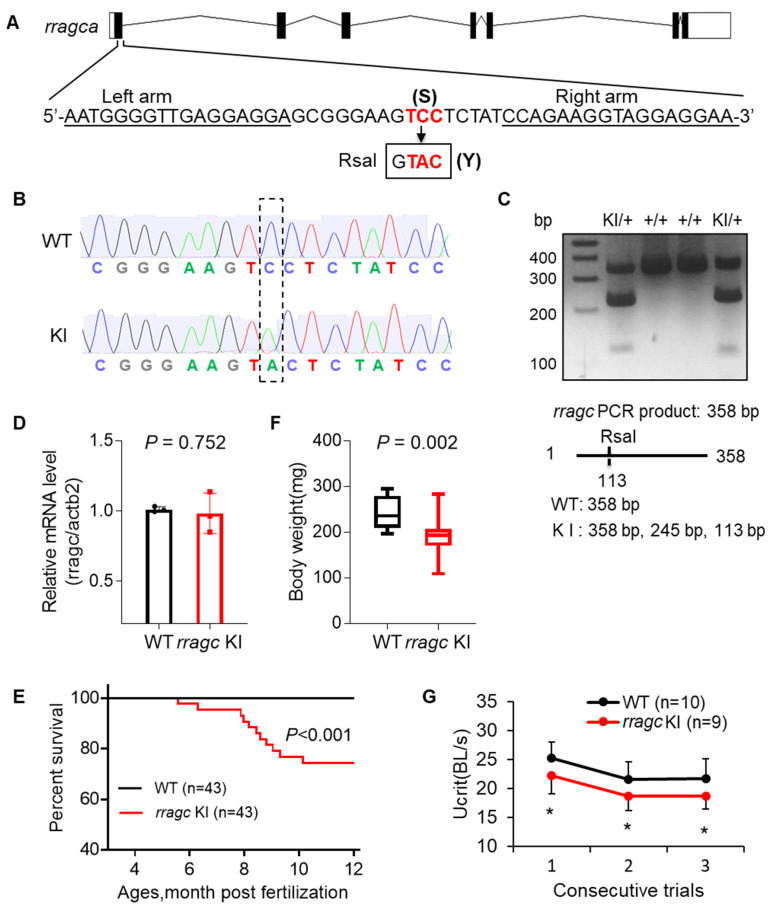
Generation of a zebrafish *rragc* S56Y knock-in mutant using TALEN. (**A**) Schematic diagram of *rragca* gene and the KI mutant. The left and right arm sequences of TALEN are underlined. The target sequence TCC (S) at residue 56 is changed to TAC (Y). Boxed is the newly created RsaI restriction enzyme recognition site. TALEN, transcription activator-like effector nucleases. (**B**) Shown are chromographs indicating the sequence change from WT to *rragc* S56Y KI homozygous. WT, wild type; KI, knock-in. (**C**) A representative genotyping gel to discern KI from WT allele. While WT allele will exhibit a 358 bp band, introduction of a new RsaI restriction site in KI results in a 245 bp and a 113 bp band. (**D**) RT-qPCR analysis of *rragc* transcript level in WT and *rragc* KI (homozygous, same in panel (**E**–**G**)) fish hearts. Data were normalized to *actb2* and expressed as fold change over WT. (**E**) Kaplan–Meier survival curves for *rragc* KI fish and WT siblings by log-rank test. (**F**) Boxplots (MIN to MAX) illustrates reduced body weight of *rragc* KI fish at the age of 7 months. *n* = 10, 19. (**G**) Swimming capacity of *rragc* KI mutant compared with the WT siblings in 3 consecutive trials at 7 months. Ucrit, critical swimming speed. BL, body length. Data in (**D**) and (**G**) are expressed as mean ± SD, Student’s t test, * *p* < 0.05 versus WT.

To determine the impact of RagC S75Y missense mutation in cardiomyopathy development, we set out to generate a corresponding zebrafish KI model. In zebrafish, there are two *RRAGC* orthologues, *rragca* and *rragcb*, encoding proteins that share 75% and 71% amino acid identity to the human RagC, respectively (Figure S1). Because the transcript of *rragca*, not *rragcb*, is predominantly expressed in the heart (Figure S2) [22], we targeted the *rragca* locus and generated a S56Y KI zebrafish via transcription activator-like effectors nucleases (TALEN) technology. A conversion of C to A was introduced, resulting in a substitution of Serine (S) by Tyrosine (Y) at amino acid 56, corresponding to the S75Y mutation in human RagC (Figure 1A). Successful genome editing was confirmed by Sanger sequencing, and the KI allele was tracked by a newly-created RsaI restriction enzyme digestion site (Figure 1B,C). We obtained Rragc^S56Y^ homozygous mutants, termed *rragc* KI for simplicity. We noted unchanged *rragca* mRNA levels in adult *rragc* KI hearts by RT-qPCR (Figure 1D). Changes in protein expression could not be assessed due to the lack of commercially available antibodies that recognize the zebrafish Rragc protein.

**Figure 2:**
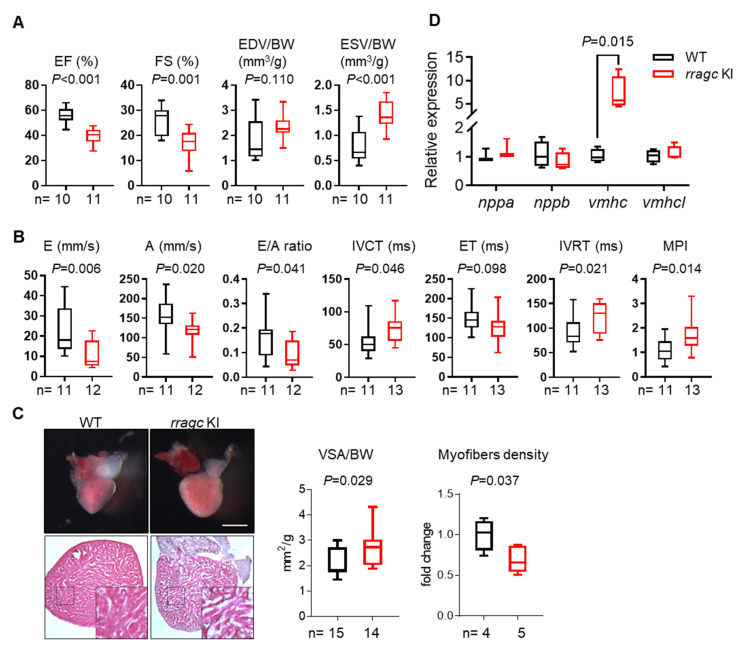
Characterizations of the cardiac phenotype of *rragc* KI fish at 7 months of age. (**A**) Echocardiographic measurements for WT (black) and *rragc* KI (red) fish. EF, ejection function. FS, fractional shortening. EDV, end-diastolic volume. ESV, end-systolic volume. BW, body weight. (**B**) High-frequency pulsed wave Doppler indices for WT and *rragc* KI fish at 7 months of age. E, early wave velocity. A, atrial wave velocity. IVCT, isovolumic contraction time. ET, ejection time. IVRT, isovolumic relaxation time. MPI, myocardium performance index, equals (IVCT + IVRT)/ET and was normalized to WT values. (**C**) Representative images of isolated hearts (upper panel) and H&E staining (lower panel), as well as corresponding quantification of the ventricular surface area (VSA) normalized to BW and trabecular muscle density in WT and *rragc* KI mutants. Scale bar = 0.5 mm. (**D**) RT-qPCR analysis of cardiomyopathy molecular markers in WT and *rragc* KI fish hearts (*n* = 4, each). Data were normalized to corresponding *actb2* levels and *rragc* KI is expressed as the fold-change versus WT. *nppa*, natriuretic peptide A; *nppb*, natriuretic peptide B; *vmhc*, ventricular myosin heavy chain; *vmhcl*, ventricular myosin heavy chain-like. Data are shown in boxplot (MIN to MAX) and analyzed by Student’s *t* test.

The *rragc* KI fish started to die at 5 months, with approximately 70% fish surviving to 1 year of age (Figure 1E). Most phenotyping studies were carried out around 7 months of age, unless otherwise indicated. The *rragc* KI fish exhibited impaired growth as indicated by lower body weight (Figure 1F). Maximum swimming capacity, a heart failure index that mimics exercise capacity in humans, was significantly reduced in the *rragc* KI mutants (Figure 1G). We noted significant ventricular systolic function decline, a hallmark of cardiomyopathy, by measuring ejection fraction (EF) and fractional shortening (FS) (Figure 2A and videos S1 and S2). This was largely ascribed to an increased end-systolic volume (ESV)/body weight (BW) ratio. The end-diastolic volume (EDV)/BW ratio remained unchanged. Impaired cardiac function was further demonstrated by pulsed-wave Doppler analysis, as indicated by the decreased E/A ratio and increased myocardial performance index (MPI) (Figure 2B). The isolated *rragc* KI hearts had enlarged ventricular surface area (VSA)/BW and reduced density of ventricular trabeculae (Figure 2C). We also assessed transcriptional changes of molecular markers of cardiac remodeling and detected significant elevation of ventricular myosin heavy chain (*vmhc*) (Figure 2D). These data confirmed pathogenicity of the S75Y variant.

### 2.2. Rragc Knockout (KO) Zebrafish Does Not Manifest Cardiomyopathy-Like Phenotypes

As byproducts of KI approach, two *rragca* KO mutant alleles were obtained (Figure S3A). M1 allele contains a 124 bp addition in exon1 that results in a premature stop codon and a 60% reduction of the *rragca* transcripts, while M2 allele contains a 10-nucleotide deletion that is also predicted to result in a premature stop codon (Figure S3A,B). Homozygous mutants for either allele did not exhibit any noticeable phenotypes until at least 1 year of age (data not shown). For example, cardiac pump function in the M1 allele remained unchanged (Figure S3C). These in vivo data demonstrated that *rragc* KI, but not *rragca* KO, recapitulated phenotypic features of cardiomyopathy as observed in the human patient with RagC^S75Y^.

### 2.3. RagC^S75Y^ Cardiomyopathy Models Exhibit Altered mTORC1–TFEB Signaling

**Figure 3:**
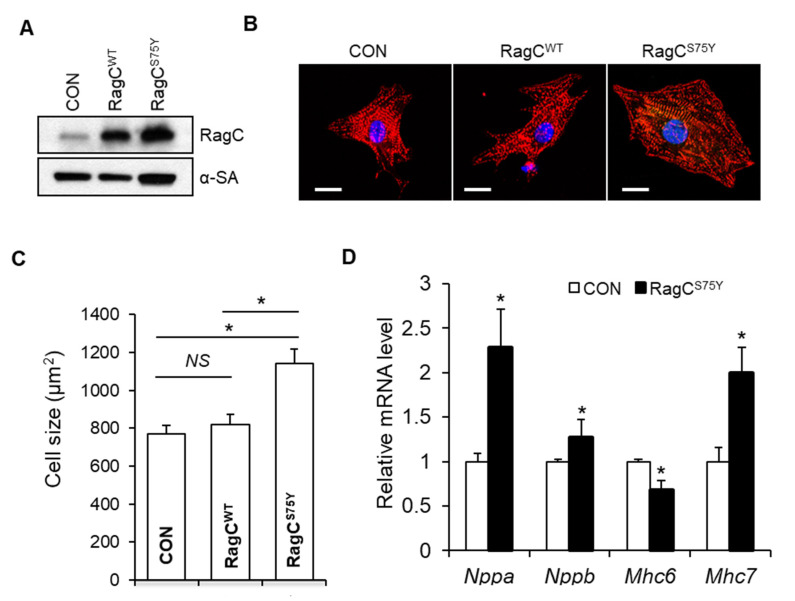
RagC^S75Y^ induces increased myocyte size in NRVCMs. (**A**) Immunoblots for RagC and alpha-sarcomeric actin (α-SA) in cell lysate of NRVCMs infected with recombinant adenoviruses including Ad:GFP (CON) Ad:RagC wild type (RagC^WT^), and Ad:RagC^S75Y^ (RagC^S75Y^). NRVCMs: neonatal rat ventricle cardiomyocytes. (**B**) Representative confocal images of NRVCMs infected for 48 h. NRVCMs were stained with an anti-alpha actinin antibody (red) and DAPI (blue). Scale bar, 20 μm. (**C**) Quantification of average cell area of three groups in (**B**). A total of 150–200 cardiomyocytes per group were measured in each experiment. Data were averaged from 3 independent experiments. * *p* < 0.05. NS, not significant. (**D**) Quantification of transcriptional level of hypertrophic molecular markers in NRVCMs. The mRNA values were normalized to 18S rRNA and expressed as fold changes over CON. *Nppa*, natriuretic peptide A; *Nppb*, natriuretic peptide B; *Mhc6*, myosin heavy chain 6; *Mhc7*, myosin heavy chain 7. *n* = 3–4. Data are represented as the mean ± SEM, * *p* < 0.05 versus CON, 2-tailed Student’s t test.

To facilitate the study of initial pathological signaling events that lead to cardiomyopathy, we turned to NRVCMs cellular models. We found that overexpression of RagC^S75Y^ via adenovirus infection for 48 h significantly increased the cell size (by 1.5 fold) when compared to control cells infected with Ad:GFP. By contrast, overexpression of wild type RagC did not affect cell size (Figure 3A–C). Because no significant difference was noticed between GFP control and RagC WT, we carried the following experiments by comparing RagC^S75Y^ with GFP control. Consistently, mRNA expression levels of hypertrophic markers such as *Nppa*, *Nppb*, and *Mhc7* were elevated in RagC^S75Y^ cells, whereas the expression of *Mhc6* was downregulated (Figure 3D). Thus, we concluded that the pathogenicity of the S75Y variant can be recapitulated in the in vitro system.

**Figure 4:**
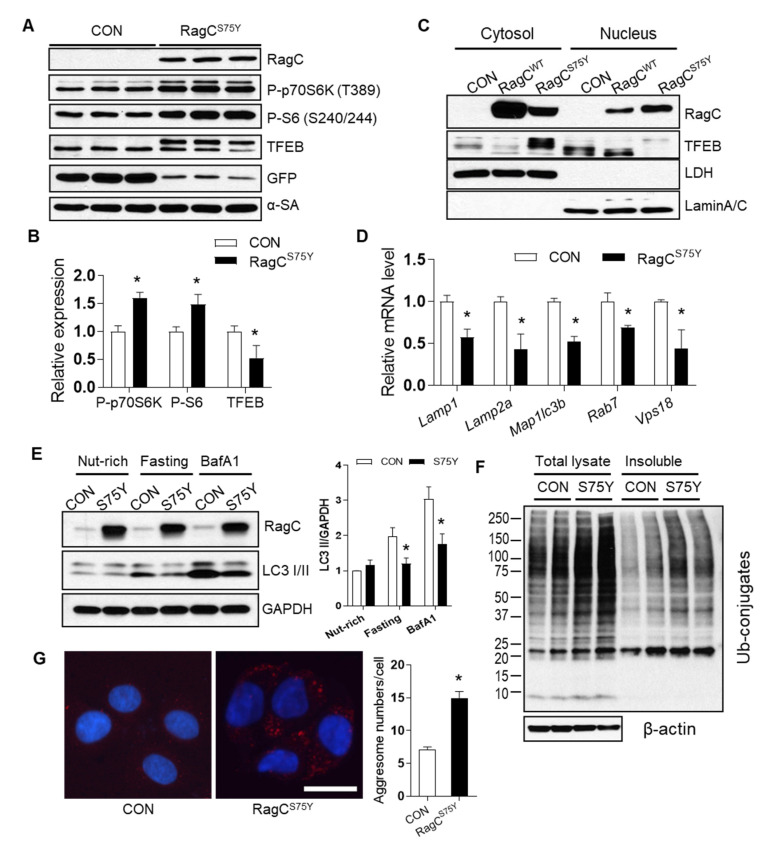
The mTORC1–TFEB-autophagy signaling was dysregulated in RagC^S75Y^ cardiomyocytes. (**A**) Immunoblot of RagC, P-p70S6K (T389), P-S6 (S240/244), TFEB, GFP, α-SA in cell lysates of NRVCMs infected with Ad:GFP (CON, same as below) and Ad:RagC^S75Y^ (RagC^S75Y^ or S75Y, same as below). (**B**) Quantification of band intensity normalized by α-SA protein level, *n* = 3. (**C**) Representative immunoblot of RagC and TFEB proteins in the nuclear and cytosolic fractions of AD293 cells transfected with GFP and RagC^S75Y^. LDH and Lamin A/C were used as cytosolic and nuclear protein loading control, respectively. (**D**) The mRNA level of TFEB target genes in NRVCMs. The mRNA values were normalized to 18S rRNA and expressed as fold change over CON, *n* = 3–4. *Lamp1*, lysosomal-associated membrane protein 1; *Lamp2a*, lysosomal-associated membrane protein 2 alpha; *Map1lc3b*, microtubule-associated protein 1 light chain 3 beta; *Rab7*, RAB7, member RAS oncogene family; *Vps18*, VPS18 core subunit of CORVET and HOPS complexes. (**E**). Representative immunoblotting images of LC3 II flux in H9C2 cardiomyocytes infected with Ad:GFP (CON) and Ad:RagC^S75Y^ (S75Y) cultured in nutrient rich (nut-rich), HBSS (Fasting, 1 h), and with Bafilomycin A1 (BafA1, 200 nmol/L, 2 h) conditions. (**F**). Total cell lysate and insoluble protein fractions of CON and S75Y cardiomyocytes were separated by SDS–PAGE and analyzed in immunoblots probed with antibodies against ubiquitin (Ub). β-actin was used as a loading control. (**G**). Representative image and quantification of aggresomes (red dots) stained by ProteoStat dye in CON and S75Y cardiomyocytes. Bar = 40 um, *n* > 100 in each group. All quantification data were shown as means ± SD (in C and D) or SEM (in E and G) and analyzed by Student’s *t* test. * *p* < 0.05 versus CON.

Consistent with the GOF hypothesis of the S75Y variant, we found hyperactive mTORC1 signaling as indicated by significantly higher phosphorylation levels of p70S6 kinase (P-p70S6K) and ribosomal protein S6 (P-S6) in S75Y-overexpressed NRVCMs when compared to GFP-infected control cells (Figure 4A,B). In contrast, TFEB expression was reduced and accompanied by an extra band with higher molecular weight (Figure 4A,B). We then revealed that the majority of TFEB in GFP- or RagC^WT^ transfected cells was in the nuclear fractions (lower band), while majority of TFEB in RagC^S75Y^ transfected cells was in the cytoplasm (upper band in Figure 4C). These data suggested that TFEB is prone to post-translational modification upon RagC^S75Y^ expression, possibly hyper-phosphorylation [13], which could account for retention in the cytoplasmic fraction. In agreement with this hypothesis, expression of several known TFEB target transcripts were significantly reduced in S75Y infected NRVCMs (Figure 4D). Because of the vital function of the Rags-mTORC1–TFEB signaling in regulating autophagy–lysosomal clearance [23,24,25], we assessed autophagy marker LC3-II. Fasting or Bafilomycin-A1 (BafA1) treatment significantly induced LC3-II levels in control cells but not in S75Y cardiomyocytes (Figure 4E), suggesting insufficient autophagy response and flux [26]. Consistently, we noted accumulation of ubiquitin-conjugated proteins in the S75Y cell lysates, especially in the insoluble fraction that is subject to autophagic degradation (Figure 4F), as well as more aggresomes (Figure 4G).

**Figure 5:**
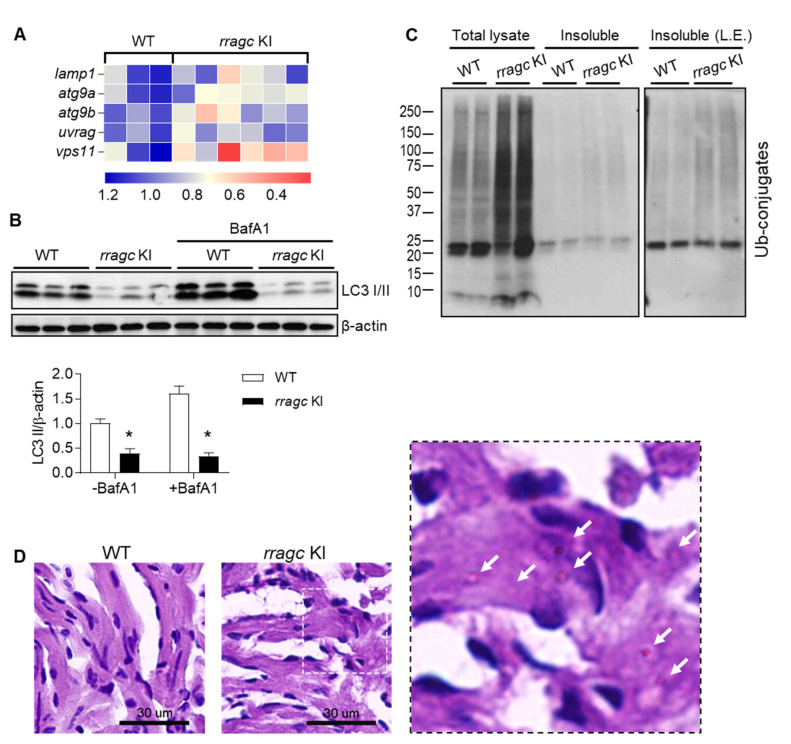
*rragc* KI fish manifests reduced TFEB activity and defective function of lysosomal clearance. (**A**) Heatmap of relative mRNA expression levels of genes regulated by TFEB in *rragc* KI and WT fish hearts. Values are shown as fold change of relative mRNA expression normalized to the mean value of WT. *actb2* was used as an internal control. *lamp1*, lysosomal associated membrane protein 1; *atg9a*, autophagy related 9A; *atg9b*, autophagy related 9B; *uvrag*, UV radiation resistance associated gene; *vps11*, VPS11 core subunit of CORVET and HOPS complexes. (**B**) Immunoblot of LC3 in heart lysates of 9-month-old *rragc* KI and WT fish treated with BafA1 (1.2 ug/g, 6 h) or vehicle via intraperitoneal injection. β-actin as a loading control. * *p* < 0.05 versus WT. (**C**) Total lysate and insoluble protein fractions of *rragc* KI and WT fish heart were separated by SDS–PAGE and immunoblotted against ubiquitin antibodies (Ub). Fish age around 1 year. L.E., longer exposure. (**D**) Periodic Acid–Schiff (PAS) staining of fish heart tissues. Representative images are shown. Scale bars, 30 μm. The right panel is the higher magnification views of sections indicated by white dash box. Arrows point to glycogen deposit.

Next, we determined whether these pathological changes also occurred in *rragc* KI fish model. Similar to the cell culture studies, transcript levels of several TFEB-controlled lysosome genes were downregulated in the *rragc* KI fish hearts (Figure 5A), suggesting a reduced TFEB activity. We also detected autophagy dysregulation, as indicated by LC3-II protein levels being severely reduced at steady state and unresponsive to BafA1 administration (Figure 5B). Elevated ubiquitin conjugated proteins in total lysate and insoluble fractions of heart tissues were also observed in *rragc* KI fish compared to wild type animals (Figure 5C). Because abnormal glycogen accumulation is the major manifestation of lysosome dysfunction as seen in the hypertrophic heart of Danon disease [27], we performed Periodic Acid–Schiff staining and observed a significant increase of glycogen deposits in *rragc* KI fish heart at 1 year of age (Figure 5D). Collectively, our data indicated that the RagC^S75Y^ cardiomyopathy is characterized with dysregulated autophagy–lysosomal clearance and glycogen deposition. At the molecular level, the primary signaling defect consists of both mTORC1 activation and TFEB inhibition.

### 2.4. mTOR Inhibition Does Not Attenuate RagC^S75Y^ Cardiomyopathy

**Figure 6:**
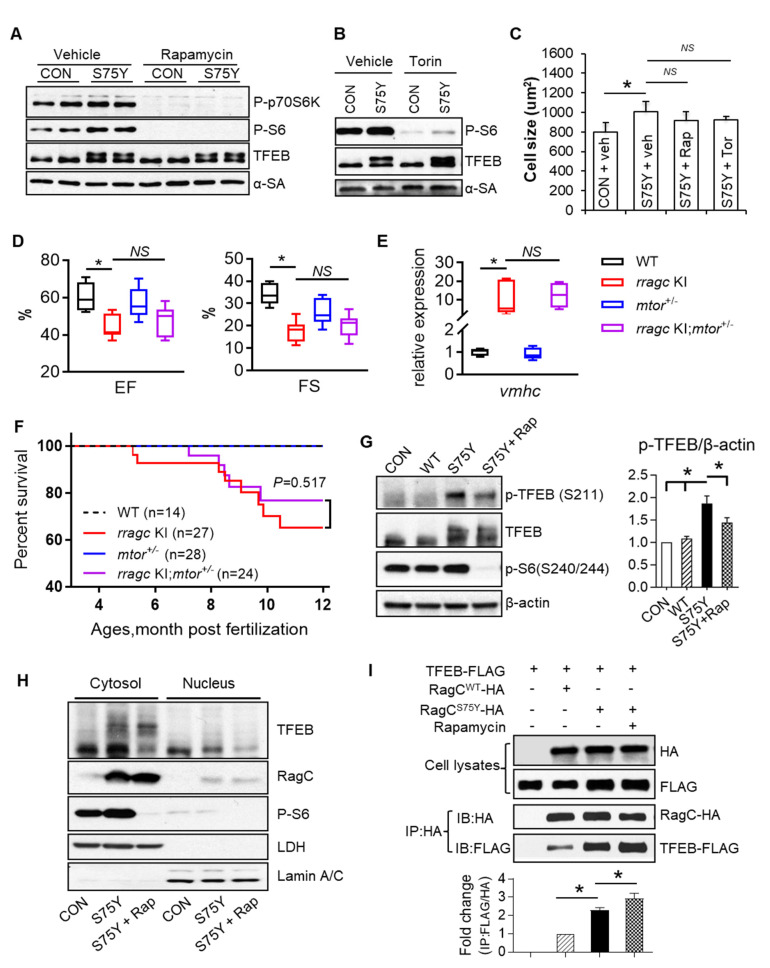
mTORC1 inhibition cannot ameliorate RagC^S75Y^ cardiomyopathy both in vitro and in vivo. (**A**,**B**) Immunoblot of P-p70S6K, P-S6, TFEB, and α-SA in NRVCMs. The NRVCMs were infected with Ad:GFP (CON) or Ad:RagC^S75Y^ (S75Y) for 24 h followed by rapamycin (100 nmol/L) or torin (10 nmol/L) or vehicle incubation for another 24 h. (**C**) Neither rapamycin nor torin rescue the enlarged cell size induced by RagC^S75Y^. Data were averaged from three independent experiments. * *p* < 0.05; NS, not significant. Data are mean ± SEM by one-way ANOVA. (**D**), *mtor* haploinsufficiency (*mtor+/−*) did not rescue reduced cardiac function in *rragc* KI fish. Shown are echocardiographic analysis of EF and FS in the WT control, single mutants, and *rragc* KI;*mtor+/−* double mutants at 7 months. *n* = 8,11,8,10. (**E**) The abnormal elevated *vmhc* mRNA expression was not attenuated in *rragc* KI;*mtor+/−* double mutant fish at 7 months compared with *rragc* KI fish. *n* = 3,5,5,5. Data in (**D**,**E**) are shown in boxplot (MIN to MAX). * *p* < 0.05, NS, not significant versus *rragc* KI, one-way ANOVA. (**F**), Kaplan–Meier survival curves of *rragc* KI;*mtor+/−* double mutant fish compared with their corresponding single mutants and the WT control by log-rank test. (**G**). Rapamycin marginally attenuated the increased phosphorylation of TFEB at S211 in S75Y cells. Quantification data was shown as mean ± SEM, * *p* < 0.05, one-way ANOVA. (**H**). Rapamycin did not promote TFEB nucleus translocation in S75Y cells. Shown are representative immunoblots of TFEB, RagC, and P-S6 proteins in the nuclear and cytosolic fractions of H9C2 cardiomyocytes infected with Ad:GFP or Ad:RagC^S75Y^ and then treated with 100 nmol/L Rapamycin (S75Y + Rap) or vehicle for 24 h. Lamin A/C and LDH were used as nuclear and cytosolic protein loading control, respectively. (**I**) AD293 cells were transfected with TFEB–FLAG and RagC^WT^-HA or RagC^S75Y^-HA; 24 h later, cells were treated with vehicle or rapamycin (100 nmol/L) or vehicle for another 24 h. Cells were lysed and subjected to immunoprecipitation with the anti-HA antibody. The immunoprecipitates and cell lysates were analyzed by immunoblotting with antibodies against HA (used to detect RagC) and FLAG (used to detect TFEB). IP: immunoprecipitation; IB: immunoblot. Quantification data mean ± SEM, * *p* < 0.05, one-way ANOVA.

To develop effective therapy for RagC^S75Y^ cardiomyopathy, we first assessed whether mTOR inhibition exerts therapeutic effects in both in vitro and in vivo models. Surprisingly, we found 24-hour treatment with mTOR inhibitors, including rapamycin and torin, failed to normalize the enlarged cell size in S75Y NRVCMs, despite drastic inhibition of mTORC1 activity as indicated by P-S6 expression (Figure 6A–C and Figure S4A). The aberrant TFEB expression, as indicated by the shifted bands, remained unchanged (Figure 6A,B). To further assess mTOR inhibition therapy by a genetic approach, we bred *mtor^xu015/+^* into *rragc* KI fish to obtain a *rragc* KI;*mtor^xu015/+^* double mutant [28]. Similarly, haploinsufficiency of *mtor* failed to exert therapeutic effect on the *rragc* KI model, as indicated by persistent cardiac pump dysfunction, unchanged cardiomyopathy marker expression, impaired growth, and reduced survival in double mutants (Figure 6D–F and Figure S4B).

These unexpected results prompted us to investigate why mTOR inhibition was insufficient to exert therapeutic effects in RagC^S75Y^ cardiomyopathy. Because the phosphorylation status of TFEB governs its cytosol-nuclear shuttling, we checked phosphorylation of S211 by mTOR, which facilitates TFEB binding with 14-3-3 protein which has been hypothesized to mask the nuclear localization signal and inhibits TFEB nuclear translocation [13]. In cells containing RagC^S75Y^, TFEB phosphorylation at S211, an mTORC1 phosphorylation site, was significantly increased, and can be effectively attenuated by rapamycin (Figure 6G), suggesting a normal mTOR–TFEB phosphorylation signaling. However, rapamycin treatment did not translocate more TFEB into nuclei (Figure 6H), suggesting an mTOR-independent regulatory mechanism. Because lysosome–cytosolic translocation of TFEB can also be controlled by active–inactive status of Rags that binds TFEB [11], we assessed binding between RagC^S75Y^ and TFEB. Co-immunoprecipitation assay in AD293 cells revealed a greater binding affinity between RagC^S75Y^ and TFEB than RagC^WT^ and TFEB (Figure 6I). Importantly, rapamycin did not attenuate Rags–TFEB interaction, nor did promote TFEB nuclear translocation (Figure 6H,I). Thus, we propose that mTOR-independent binding between RagC^S75Y^ and TFEB renders mTOR inhibition ineffective.

### 2.5. TFEB Activation Attenuates RagC^S75Y^ Cardiomyopathy

**Figure 7:**
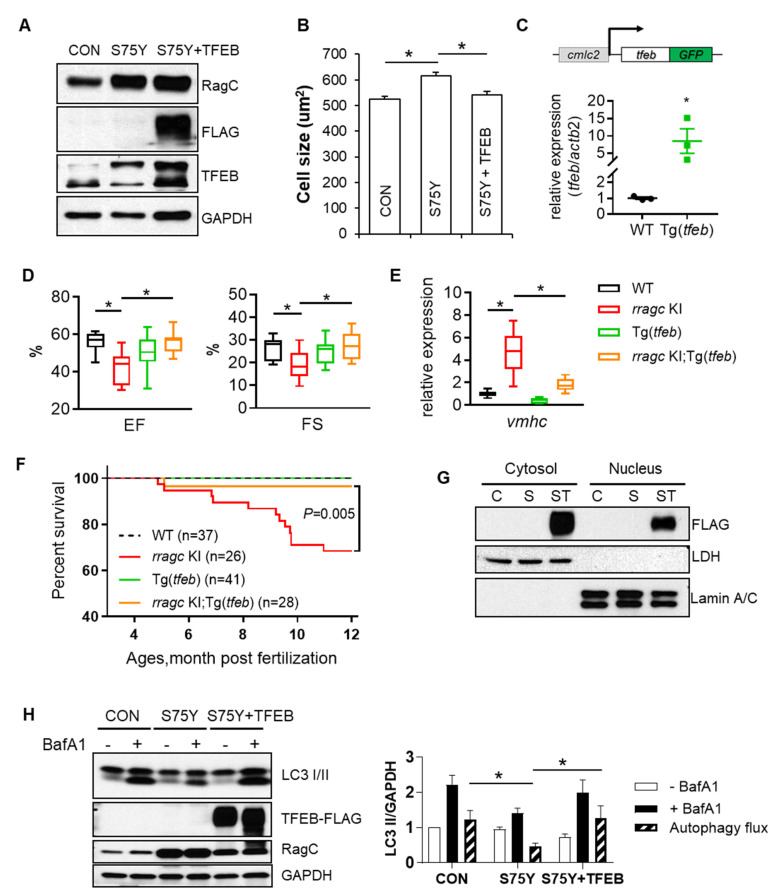
Ectopic expression of TFEB ameliorates RagC^S75Y^ cardiomyopathy both in vitro and in vivo. (**A**) Immunoblots for RagC, TFEB, and α-SA in cell lysate of NRVCMs infected with recombinant adenoviruses, Ad:GFP (CON), Ad:RagC^S75Y^ (S75Y), Ad:TFEB–FLAG (TFEB) for 48 h. (**B**) Ectopic TFEB expression normalized cell size in S75Y NRVCMs. NRVCMs were stained for alpha actinin for measurement of cell surface area. A total of 150–200 cardiomyocytes per group were measured in each experiment. Data were averaged from 3 independent experiments. * *p* < 0.05; NS, not significant. Data are mean ± SEM by one-way ANOVA. (**C**) Schematic diagram of the cardiac-specific *tfeb* transgenic fish (upper panel) and their relative *tfeb* mRNA expression compared to WT fish. * *p* < 0.05, data are mean ± SEM by student’s t test. (**D**) Cardiac-specific TFEB overexpression by a transgenic line (Tg*(tfeb*)) rescued cardiac function in *rragc* KI fish. Shown are echocardiographic analysis of EF and FS in *rragc* KI;Tg(*tfeb*) double mutant fish at 7 months compared with their corresponding single mutants and the WT control. *n* = 10,12,10,13. (**E**) Ectopic TFEB expression rescued cardiomyopathy molecular marker expression in vivo. Shown are relative mRNA expression of *vmhc* normalized to actb2 by RT-qPCR in *rragc* KI;Tg(*tfeb*) double mutant fish at 7 months compared with their corresponding single mutants and the WT control. *n* = 3,6,3,6. Data in (**D**) and (**E**) are shown in boxplot (MIN to MAX). * *p* < 0.05, NS, not significant versus *rragc* KI, one-way ANOVA. (**F**) Ectopic TFEB expression improved survival. Shown are Kaplan–Meier survival curves of *rragc* KI;Tg(*tfeb*) double mutant fish compared with their corresponding single mutants and the WT control during 1 year observation by log-rank test. (**G**) Overexpressed TFEB–FLAG is able to translocate into nuclei. Shown are immunoblots of FLAG indicating exogenous TFEB proteins in the nuclear and cytosolic fractions of CON (C), S75Y (S) and S75Y + TFEB (ST) H9C2 cardiomyocytes. Lamin A/C and LDH were used as nuclear and cytosolic protein loading control, respectively. (**H**) Ectopic TFEB expression was sufficient to restore dysregulated autophagy flux in S75Y cells. Shown are representative immunoblotting images in CON, S75Y, and S75Y + TFEB cardiomyocytes treated with BafA1 (200 nM, 2 h) or vehicle. LC3 II band intensity normalized by GAPDH were quantified in the graph. Autophagy flux were calculated as “black column value”−“white column value”, *n* = 4. * *p* < 0.05, data are mean ± SEM by one-way ANOVA.

We then tested whether TFEB could be a therapeutic target for RagC^S75Y^ cardiomyopathy. Consistent with this hypothesis, ectopic expression of TFEB was able to normalize NRVCM size that was enlarged by RagC^S75Y^ (Figure 7A,B and Figure S5A). Next, we generated Tg(*cmlc2:tfeb-GFP*), a transgenic fish line that drives cardiomyocyte-specific *tfeb* expression 9-fold higher than wild type control (Figure 7C). Indeed, the transgene was sufficient to attenuate cardiomyopathy phenotypes in the *rragc* KI model, as indicated by restoration of EF, FS, and *vmhc* expression in *rragc* KI;Tg*(tfeb)* fish compared to age matched *rragc* KI alone at 7 months (Figure 7D,E). The overall survival of *rragc* KI;Tg*(tfeb)* fish was significantly improved compared to *rragc* KI mutants (Figure 7F), despite marginal effect on bodyweight (Figure S5B). At the molecular level, we noted considerable amounts of exogenous TFEB in the nuclei of RagC^S75Y^ cardiomyocytes (Figure 7G). As a consequence, impairment of autophagy flux that was resulted from S75Y expression was restored (Figure 7H). In summary, we concluded that TFEB activation, but not mTOR inhibition, is therapeutic to RagCS75Y cardiomyopathy.

## 3. Discussion

### 3.1. Molecular Mechanisms of RagC^S75Y^ Cardiomyopathy

In this manuscript, we conducted comprehensive functional studies of RagC^S75Y^, a candidate pathogenic variant that was recently identified in a pediatric DCM patient [4]. Extensive evidence from studies using both in vivo animal models and primary cardiomyocytes prove the RagC^S75Y^ variant as pathogenic for cardiomyopathy. First, zebrafish mutants harboring Rragc^S56Y^ KI manifested cardiomyopathy-like phenotypes, including decreased cardiac function, ventricular remodeling, reduced exercise capacity, and reduced survival. Second, overexpression of RagC^S75Y^ caused cellular hypertrophy in NRVCMs. Our data also confirmed the GOF nature of the S75Y variant. For instance, *rragca* KO zebrafish did not exhibit cardiomyopathy-like phenotypes; the Rags complex in NRVCMs appeared activated that was supported by increased S6K/S6 phosphorylation, impaired TFEB nuclear-translocation, and decreased TFEB transcriptional activity. Importantly, we uncovered that the S75Y substitution significantly increases the binding between Rags and TFEB, and subsequently affects the nuclear translocation of TFEB. This is likely a key pathological event that ultimately leads to cardiomyopathy.

Besides cardiomyopathy, RRAGC has been previously reported to be an important genetic factor for follicular lymphoma. Recurrent somatic mutations in *RRAGC* were noted in 17% patients with follicular lymphoma [15,29]. The mutations were predominantly missense that clustered around the nucleotide-binding domain, with amino acid S75 as one of the hotspots [15]. Most of these RagC variants led to increased binding to Raptor and enhanced mTORC1 signaling even under nutrient deprivation, which eventually accelerated lymphomagenesis [15,16]. It remains to be determined whether follicular lymphoma occurs in RagC^S75Y^ cardiomyopathy patients, and if so, whether and why cardiomyocytes and lymphocytes are more susceptible than the other organs to pathological damages incurred by RagC GOF.

**Figure 8:**
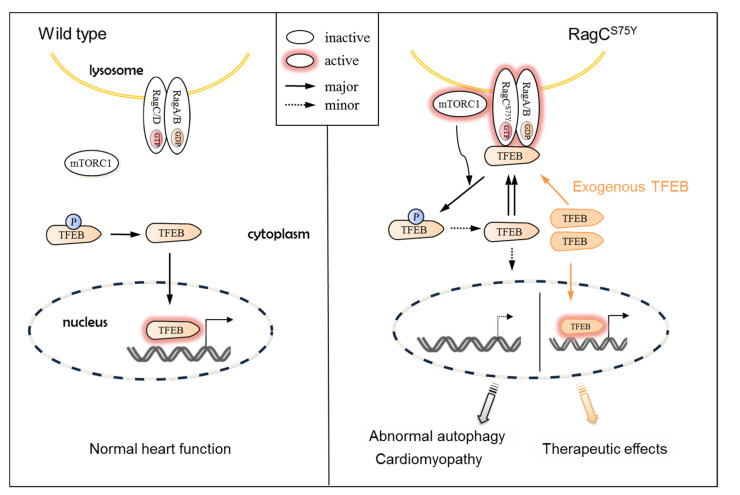
Schematic mechanism of RagC^S75Y^ cardiomyopathy and a candidate therapeutic strategy. (**Left**) Under full nutrients condition, the wild type heterodimeric Rags (formed by either RagA or RagB and RagC and RagD) is in the inactive state, leading to mTORC1 detachment from the lysosome and to its inactivation. TFEB is dephosphorylated and able to translocate to the nucleus, where it activates gene expression programs that boost lysosomal function and autophagy. (**Right**) The S75Y mutation drives Rags to be active, which recruits mTORC1 and TFEB to the lysosome surface, where phosphorylation of TFEB by active mTORC1 happens. The TFEB cycles between cytoplasm and the lysosomal surface limits its nucleus translocation, which triggers cascades of pathological changes, such as dysregulated autophagy, which ultimately result in cardiomyopathy. Exogenous expression of TFEB is an effective therapeutic strategy that restores nucleus translocation of TFEB and ameliorates RagC^S75Y^ cardiomyopathy.

While our functional studies of RagC^S75Y^ demonstrated that Rags complex GOF leads to cardiomyopathy, a recent study of a cardiac-specific KO of both RagA and RagB suggested that Rags complex loss-of-function (LOF) also causes cardiomyopathy in mice [30]. We did not observe any cardiac phenotypes in *rragca* KO fish, probably because of the compensation from *rragd*. Of note, genetic evidence linking Rags LOF to human cardiomyopathy are currently lacking. Notwithstanding, it is intriguing to note that both GOF and LOF mutations at the Rags complex resulted in cardiomyopathy. In contrast to Rags GOF that prevented TFEB nuclear translocation by enhancing the Rags–TFEB interaction (Figure 8), Rags LOF impaired lysosome acidification by damaging v-ATPase activity independent of hyperactive TFEB activity [30]. Despite different mechanisms, both models share defective autophagy–lysosomal clearance and deposition of glycogen [30]. These observations underscore the importance and complexity of the Rags complex in maintaining cardiac health—disturbance of this complex in either direction could trigger cascades of pathological changes that ultimately lead to cardiomyopathy.

### 3.2. TFEB, Not mTOR, Could Be the Therapeutic Target for RagCS75Y Cardiomyopathy

The establishment of an in vitro cell culture model and an in vivo animal model of RagC^S75Y^ cardiomyopathy laid the foundation for mechanistic studies and therapy development. We detected an aberrant Rags–mTOR–TFEB signaling that is characterized by excessive mTORC1 signaling and defective TFEB function. We did not provide direct evidence to support that TFEB deficiency solely and specifically causes the phenotypes. Nevertheless, we conducted both in vitro and in vivo studies that consistently identified TFEB overexpression as an effective therapeutic avenue. This could be at least partially explained that exogenous TFEB alters the stoichiometry of Rags-bound vs. cytosolic free TFEB and, hence, sufficient TFEB can translocate into the nuclei to activate downstream signaling (Figure 8). TFEB overexpression could be a translatable therapeutic avenue for RagC^S75Y^ cardiomyopathy, because adeno-associated gene transfer system has been proven safe in humans [31,32]. Moreover, screening of compounds that enhance TFEB nuclei translocation via directly activating TFEB or antagonizing Rags–TFEB interaction could lead to additional therapeutic avenues for RagC^S75Y^ cardiomyopathy.

Both of our in vitro and in vivo studies consistently demonstrated that mTOR inhibition cannot exert therapeutic effects for RagC^S75Y^ cardiomyopathy. The observation was initially surprising, because mTOR inhibition has been shown to have broad applicability to many types of cardiomyopathies [18,28,33,34] and mTORC1 signaling was obviously activated in our RagC^S75Y^ cardiomyopathy models. However, given that RagC^S75Y^ cardiomyopathy could be ascribed to the increased Rags–TFEB binding that is independent of mTOR signaling, mTOR inhibition is also possible not to exert therapeutic benefits. On the other hand, a similar GOF mutation of RagC S75C accelerates lymphomagenesis and responds to rapamycin treatment [16]. It is possible that the same RagC GOF variant incurs different pathological responses in lymphocytes versus cardiomyocytes—while mTOR-associated growth and survival are the critical mechanism underlying lymphomagenesis [16], these events might be less essential in cardiomyocytes with little proliferation capacity.

### 3.3. Zebrafish Can Be Used for Studying Genetic Variants Identified in Human Cardiomyopathy

Zebrafish is the simplest vertebrate model for cardiomyopathy—instead of four-chambered hearts in mammals, a zebrafish heart consists of two chambers. While the adult zebrafish has been pursued as an alternative vertebrate model for human diseases because of its fecundity, efficient genetics and economic husbandry, it remains unclear whether this lowly animal model possesses sufficient sensitivity to develop precision medicine for cardiomyopathies of different etiology. Adult zebrafish models of inherited cardiomyopathy have been recently established by using genome editing-based KO technologies and/or transgenic approaches [34,35,36]. KI models for cardiovascular diseases also became feasible [37,38,39]. To the best of our knowledge, Rragc^S56Y^ is the first adult zebrafish KI cardiomyopathy model. Our study demonstrated that the simple zebrafish model possesses sufficient sensitivity to recapitulate cardiomyopathy disease pathogenesis incurred by a single nucleotide change. The KI technology is essential for these types of cardiomyopathies, because KO models could yield misleading information. Thus, our data endorse the use of adult zebrafish as a sensitive model to test and validate variants of unknown significance identified in clinical genetic testing.

The present study also highlights the potential of adult zebrafish for developing precision medicine for cardiomyopathy. We recently generated a *bag3* (BCL2 associated athanogene 3) DCM model and a *lamp2* (lysosomal-associated membrane protein 2) hypertrophic cardiomyopathy (HCM) model [33,34], demonstrating that different types of inherited cardiomyopathies can be created in this efficient vertebrate model. Here, we report that while mTOR inhibition is effective in treating both *bag3* DCM model and *lamp2* HCM model [33,34], it fails to exert therapeutic effects on the RagC^S75Y^ cardiomyopathy. Instead, TFEB activation is effective for this particular type of cardiomyopathy. Our data justify future efforts to generate zebrafish models for cardiomyopathies of different etiologies, to decipher underlying mechanisms and develop individualized therapies.

## 4. Materials and Methods

### 4.1. Production of Rragc Knock-in Fish

Zebrafish were maintained under a 14 h light/10 h dark cycle at 28.5 °C and handled with care. Transcription activator-like effector nuclease (TALEN) techniques were employed to generate *rrgac* knock-in mutant fish as previously published [40]. Briefly, using Zifit. Available online: http://zifit.partners.org/ZiFiT/ChoiceMenu.aspx (accessed on 21 May 2021), TALEN targeting sequences were selected in the first exon of *rragca* gene: The TALEN left arm binding sequence, 5′- AATGGGGTTGAGGAGGA-3; right arm binding sequence 5′-CCAGAAGGTAGGAGGAA-3′. Both TALEN arms were then assembled using a Golden Gate kit (Addgene) [41]. The corresponding mRNAs were synthesized using mMESSAGE mMachine T3 (Ambion). About 25 pg capped mRNA were injected with single stranded donor DNA (ATCCTGCTAATGGGGTTGAGGAGGAGCGGGAAGTACTCTATCCAGAAGGTAGGAGGAATATGTGATATTA) into 1-cell–stage embryos of zebrafish (Danio rerio) WIK. Founder fish (F0) were raised to adulthood and outcrossed to give F1 embryos. Individual F1 embryos were used for genotyping PCR to identify knock-in mutant. Forward primer: 5′-CGGCGTATAAAGAATTGCTGG-3′, and reverse primer: 5′-GTGAAGTAGGTGAGCAAGAC-3′ were used for genotyping PCR. Resultant PCR products were digested with restriction enzyme RsaI I to determine genotype for wild type or Knock-in. The mutagenized nucleotide sequence of knock-in fish was confirmed by Sanger sequencing.

### 4.2. Production of Transgenic Fish Lines

To generate the fish lines Tg *(cmlc2:tfeb–egfp)*, the Tol2-based expression vectors were co-injected with 100 ng/μL transposase into zebrafish embryos at the one-cell stage. Fish with EGFP-positive heart were selected to generate founder fish. Stable transgenic lines were identified by outcrossing founder fish and screening for EGFP-positive embryos.

### 4.3. Cell Culture

Neonatal Rat Ventricle Cardiomyocytes (NRVCMs) were prepared from 2- day-old Wistar rat pups (Envigo, Indianapolis, IN, USA) using enzymatic digestion method. Briefly, ventricular tissue was minced into smaller pieces and dissociated by collagenase II (Worthington Biochemical Corp.)/pancreatin (Sigma-Aldrich, St. Louis, MO, USA) mixture. The resulting cell culture was enriched for cardiomyocytes through a 1-hour pre-plating step. The primary cardiomyocytes were cultured for 24 h in DMEM/M199 medium in the presence of 10% horse serum, 5% fetal bovine serum, 100 U/mL penicillin/streptomycin and 100 µM bromodeoxyuridine. The medium was then removed and cardiomyocytes were serum-starved for 24 h before adenoviral infection. H9C2 cells (ATCC, Manassas, VA, USA) and AD293 cells (Agilent Technologies, Santa Clara, CA, USA) were cultured in DMEM medium in the presence of 10% FBS and 100 U/mL penicillin/streptomycin (Gibco, New York, NY, USA).

### 4.4. Production of Recombinant Adenovirus

The AdEasy Adenoviral vector system (Agilent Technologies, Santa Clara, CA, USA Catalog #240031 and #240032) was used to generate recombinant adenoviruses. Full length cDNA of human WT or S75Y RagC was subcloned into the pshuttle vector, pshuttle–IRES–hrGFP under the control of CMV promoter. The constructed vector was co-transformed into BJ5183 competent cells (Agilent Technologies, Santa Clara, CA, USA) together with pAdEasy-1, the viral DNA plasmid. Recombinant Adenovirus plasmid DNA was generated via homologous recombination and then transfected AD293 cells with lipofectamine 2000 (Thermo Fisher, Waltham, MA, USA) to produce recombinant adenoviruses, Ad:RagC^WT^ and Ad:RagC^S75Y^. The Ad:GFP was used as a control.

### 4.5. Measurement of Cell Size

Neonatal cardiomyocytes grown on gelatin-coated chamber slides were infected with recombinant adenovirus for 48 h. Cardiomyocytes were fixed in 4% formaldehyde for 15 min, permeabilized with 0.2% Triton X-100 in PBS for 15 min and blocked by incubation in 5% BSA solution for 1 h at room temperature. Cardiomyocytes were stained with the antibody against α-actinin (Sigma-Aldrich, St. Louis, MO, USA, #A7811) and Alexa Fluor 568 conjugated secondary antibody (Life Technologies, Carlsbad, CA, USA). Images were acquired by confocal laser microscope (Zeiss LSM780 confocal microscope). The surface areas were measured using NIH image software (Image J). About 150–200 individualized cells were analyzed for each experiment.

### 4.6. Preparation of Protein Extracts and Immunoblotting

Protein samples were prepared from treated cells using lysis buffer containing protease inhibitor (Sigma-Aldrich, St. Louis, MO, USA). Nuclear and cytosolic fractions were prepared according to instructions of NE-PER extraction kit (Pierce, Rockford, IL, USA). Total lysate, soluble and insoluble fractions were prepared as previously described [42]. Equal amount of protein lysates were loaded and separated in a SDS–PAGE gel, which was transferred onto a polyvinylidene difluoride membrane (Bio-Rad Laboratories, Hercules, CA, USA). Membranes were blocked in 5% non-fat milk and incubated with primary antibodies overnight at 4 °C. The following primary antibodies were used: RagC (#5466), Phospo-p70 S6 kinase (T389, #9234), Phospho-S6 ribosomal protein (S240/244, #2215), LDHA (#3582), Lamin A/C (#4777), phospho-TFEB(Ser211, #37681S), FLAG (#2044) from Cell Signaling Technology, Danvers, MA, USA, α-sarcomeric actin (Sigma-Aldrich, St. Louis, MO, USA, #A2172), β-actin (Sigma-Aldrich, St. Louis, MO, USA, #A3854), GFP (Agilent Technologies, Santa Clara, CA, USA, #240141), LC3 (Novus Biologicals, Littleton, CO, USA, #NB100-2331), TFEB (Bethyl Laboratories, Montgomery, TX, USA, #A303-673A-M), ubiquitin (Thermo Fisher, Waltham, MA, USA, #PA5-17067), GAPDH (Santa Cruz, #sc-25778), HA.11 (16B12) (Covance, Princeton, MJ, USA, #MMS-101P). Membranes were incubated with appropriate secondary antibodies conjugated to horseradish peroxidase (HRP) (Santa Cruz Biotechnology, Dallas, TX, USA) and signals were visualized by Chemiluminescence (GE Healthcare, Chicago, IL, USA). Three independent experiments were conducted and densities of the immunoreactive bands were evaluated using NIH Image software. Statistical analysis was performed using student’s t-test or one-way ANOVA.

### 4.7. Coimmunoprecipitation

The plasmid constructs expressing TFEB–FLAG, together with HA-tagged RagC^WT^, or RagC^S75Y^ were transfected into AD293 cells using Lipofectamine 2000 (Thermo Fisher) or FuGENE6 (Promega, Madison, WI, USA). Twenty four hours after transfection, cells were treated with fresh growth medium containing rapamycin or vehicle for another 24 h. Cells were washed with ice-cold PBS, resuspended in NP40 lysis buffer (10 mM Na phosphate, pH 7.2, 150 mM NaCl, 1% NP-40, 2 mM EDTA, 1 mM EGTA, 20 mM NaF, 1 mM Na_3_VO_4_, 20 mM Glycero-2-phosphate) in the presence of protease inhibitor cocktail. After a 30-min incubation on ice, cell lysates were transferred into pre-washed HA probe (Santa Cruz Biotechnology, Dallas, TX, USA, #sc-7392 AC) and incubated on a shaking bed overnight at 4°C. The immunoprecipitates were washed and boiled in protein loading buffer. The total cell lysate and immunoprecipitates were subjected to western blots to detect HA and FLAG.

### 4.8. Aggresome Staining

H9C2 cells were cultured in the 8 well slide chamber and processed to adenovirus infection to overexpress RagCS75Y or GFP control for 48 h. Aggresome staining were performed according to instructions of PROTEOSTAT ^®^ Aggresome detection kit (Enzo, Farmingdale, NY, USA). Images were acquired by EVOS FL auto Imaging System (Thermo Fisher, Waltham, MA, USA). Aggresomes were counted and more than 100 individualized cells were analyzed in each group.

### 4.9. Autophagy Flux

H9C2 cells were infected with Ad:GFP or Ad:RagC^S75Y^ and cultured in nutrient rich condition as control, in Hank’s balanced salt solution (HBSS) for 1 h to induce autophagy, or with Bafilomycin A1 (BafA1, 200 nmol/L) incubation for 2 h to block autophagy. Cells were harvested for protein analysis. Seven-month-old zebrafish received BafA1 (1.2 ug/g) or vehicle administration via intraperitoneal injection 24 h later from the last feeding. Six hours later, fish hearts were harvested for protein analysis. Quantification of autophagy flux was performed as previously published [43]. Briefly, the autophagy flux value in each group was calculated as relative value of LC3 II in the presence of BafA1 minus LC3 II value of vehicle treated.

### 4.10. RNA Isolation and RT-PCR

Total RNA was isolated from cell or heart tissue using TRIzol reagent (Life Technologies, Carlsbad, CA, USA), and cDNA synthesized using Superscript III reverse transcriptase and random hexamers according to manufacturer’s guidelines (Life Technologies, Carlsbad, CA, USA). Real time PCR was performed using SYBR green (Bio-Rad, Hercules, CA, USA) using primers listed in Table S1. Ct values of targeted mRNA were normalized to the Ct values of 18S rRNA or actb2. Relative expressions of these genes were calculated by the 2^−ΔΔCT^ method.

### 4.11. Zebrafish Echocardiography

Transthoracic echocardiography was performed in zebrafish using the Vevo 3100 high-frequency imaging system (FUJIFILM VisualSonics Inc., Toronto, ON, Canada) as previous described [44]. Briefly, Adult zebrafish were anesthetized in tricaine (0.02%) for 5 min, placed ventral side up, and held in place with a soft tricaine immersed sponge stage. The 50 MHz (MX700) linear array transducer covered with acoustic gel (Aquasonic^®^ 100, Parker Laboratories, Inc., Fairfield, NJ, USA) was placed above the zebrafish to provide a sagittal imaging plane of the heart. B-mode images were acquired and then analyzed using the VevoLAB workstation. The representative movies of WT and *rragc* KI fish heart were provided as the videos S1 and S2. Ventricle area (A), long axis’s length (L) at end-diastole, and end-systole were measured. End-diastolic volume (EDV) and end-systolic volume (ESV) were calculated using a single-plane formula: V = 8 A^2^/3πL. Ejection fraction (EF) = (EDV − ESV)/EDV*100%. FS = (Ld − Ls)/Ld*100%, whereby Ld and Ls denote ventricle lengths at end-diastole and end-systole, respectively. For each index on individual fish, measurements were performed on three independent cardiac cycles to acquire average values.

Pulsed-wave Doppler (PWD) analysis was performed using our reported Doppler imaging technique [45]. Briefly, under the guidance of B-mode imaging, a Doppler gate (window) was positioned downstream from the atrioventricular (AV) valve in the ventricular inflow region to interrogate inflow velocities. The estimated Doppler angle was set parallel to red blood flow signal, and the pulse repetition frequency was 9.5 kHz. PWD signals were recorded for ~10 s for each fish and stored for off-line analysis. The following indices were measured using VevoLAB: the heights of E wave (early wave velocity) and A wave (atrial wave velocity), and the lengths of isovolumic contraction time (IVCT), ejection time (ET) across the ventriculo-bulbar valve, and isovolumic relaxation time (IVRT). E/A ratio was calculated to indicate diastolic function and myocardial performance index (MPI) was calculated as follows: MPI = (IVCT + IVRT)/ET, which constitutes an integrated measure of systolic and diastolic function.

### 4.12. Measurement of Ventricular Surface Area to Body Weight Index

Heart sizes of adult zebrafish were conducted as we previously described. To measure body weight (BW), fish were anesthetized in tricaine (0.02%) solution for 5 min, semidried on a paper towel and weighed on a scale. Then the zebrafish hearts were dissected and immersed into PBS buffer till cardiac arrest. Individual heart was imaged next to a millimeter ruler under a Leica MZ FLI III microscope. The largest projection of a ventricle, namely ventricle surface area (VSA), was outlined and measured based on the scale of the millimeter ruler using the ImageJ (NIH) software. Final data were shown as VSA/BW.

### 4.13. Histology

Hearts were harvested from adult fish at the designated stages after being euthanized by incubation with 0.02% tricaine for 5 min. Dissected tissues were immediately fixed in 4% PBS-buffered formaldehyde and sent to the Mayo Clinic Histology Core Laboratory for subsequent sample processing and hematoxylin–eosin (H&E) staining. Periodic acid–Schiff (PAS) staining were performed according to instructions of the manufacturer (Sigma-Aldrich, St. Louis, MO, USA #101646). Images were captured using the EVOS FL Auto Imaging System (Thermo Fisher, Waltham, MA, USA). The density of trabecular muscle was quantified using ImageJ software and calculated as area of myofibers divided by area of the whole ventricle wall (exclude ventricle chamber).

### 4.14. Swimming Tunnel Assay

The swimming capacity assay of adult zebrafish was conducted using a swim tunnel respirometer (Mini Swim 170, Loligo Systems, Viborg, Denmark). Testing protocol was derived from previous publications with modifications [46]. The swimming water was controlled under constant temperature around 28.0°C with continuous oxygen supply through air stone. Adult fish (Nmax = 10) were placed into the swimming tunnel with an initial water flow at 3 cm/s for a 20-minute acclimation. Water flow was then gradually increased by 5.72 cm/s (Ti) every 150 s (Tii) until all fish were exhausted. The values of speed at the last stage (Uii) and the previous stage (Ui) were recorded for each individual fish. The critical swimming capacity (Ucrit) was calculated with the following formula: Ucrit = Ui + (Uii*(Ti/Tii)). Ucrit was then normalized to an average body length in the group (BL). The same batches of fish were tested in triplicate daily.

### 4.15. Statistics

Data analyses were conducted using GraphPad Prism 8.0 (GraphPad Software), with a 2-tailed Student’s t tests or 1-way ANOVA followed by a post hoc Tukey–Kramer test. Survival data were performed using Kaplan–Meier survival analysis with a log-rank statistical method. A *p* < 0.05 was considered statistically significant.

## Supplementary Materials

The following are available online at https://www.mdpi.com/article/10.3390/ijms22115494/s1, Figure S1: Alignment of the human RRAGC and zebrafish Rragc proteins. Figure S2: *rragca* is more predominantly expressed than *rragcb* in zebrafish heart. Figure S3: *rragca* knockouts do not manifest cardiac phenotypes. Figure S4: Effects of mTOR inhibition on phenotypes of RagC^S75Y^ cardiomyopathy. Figure S5: Effects of TFEB activation on phenotypes of RagC^S75Y^ cardiomyopathy. Video S1: Representative echocardiographic video of WT fish. Video S2: Representative echocardiographic video of *rragc* KI fish. Table S1: Primers for qPCR and genotyping.
