# Supplementary material for: TFEB Overexpression, Not mTOR Inhibition, Ameliorates RagCS75Y Cardiomyopathy
Source: Int J Mol Sci. 2021 May 23;22(11):5494. doi: 10.3390/ijms22115494 (PMC8197163; doi:10.3390/ijms22115494)
Supplement: Supplementary file 1 [file ijms-22-05494-s001.zip › supplementary Figures and tables.pdf]

Supplementary Figures

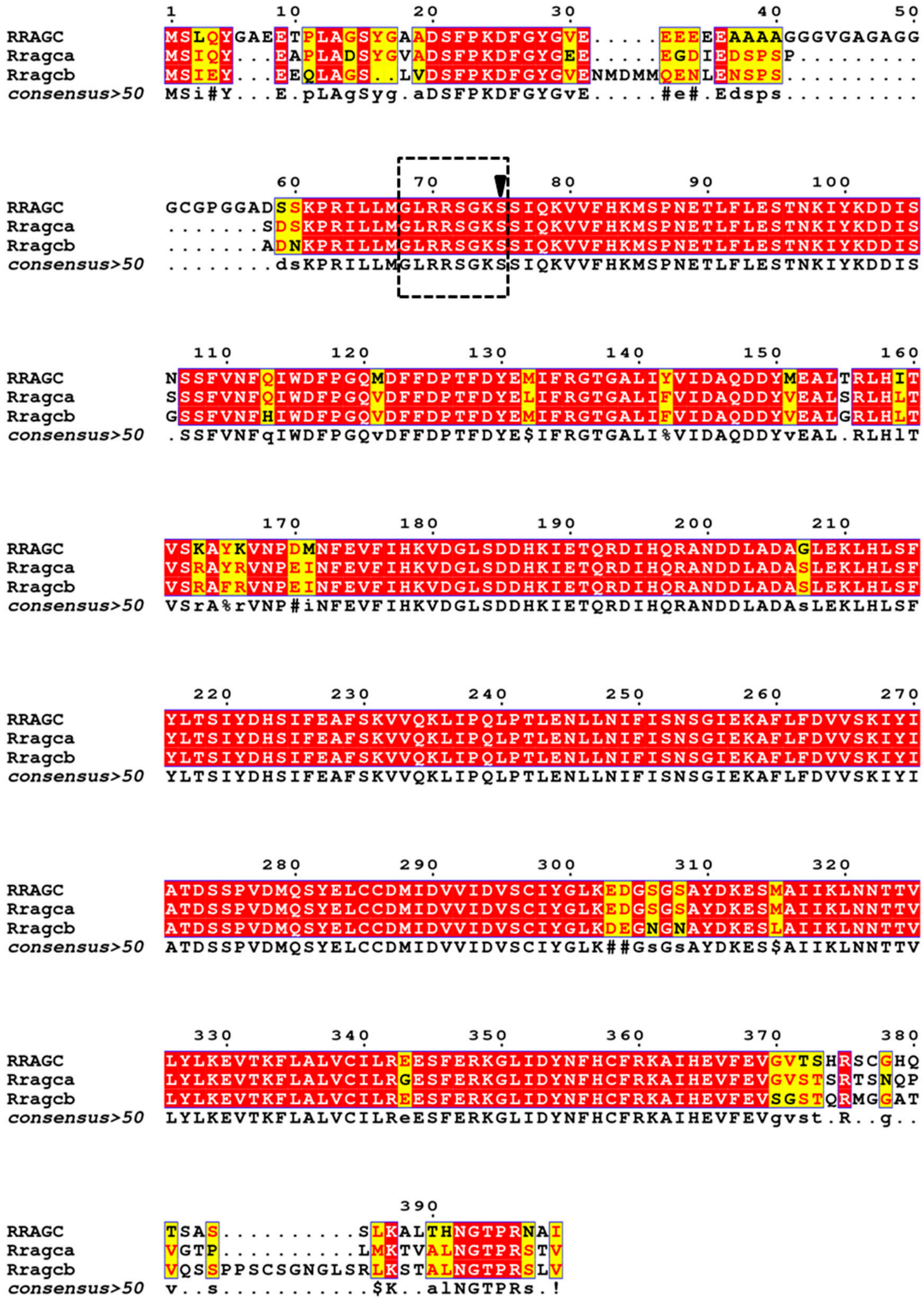

Figure S1. Alignment of the human RRAGC and zebrefish Rragc proteins. In zebrafish, there are two *RRAGC* orthologs, *rragca* and *rragcb*, encoding proteins that share 75% and 71% amino acid identity to the human RRAGC, respectively. S75 residue was indicated by an arrowhead and the P-loop motif was boxed.

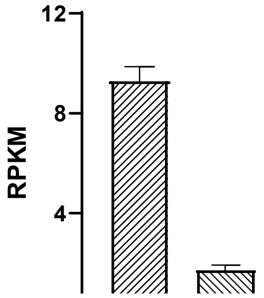

Figure S2. *rragca* is more predominantly expressed than *rragcb* in zebrafish heart. Shown are RNA-seq analysis of 6-months-old adult zebrafish. RPKM, reads per kilobase per million reads.

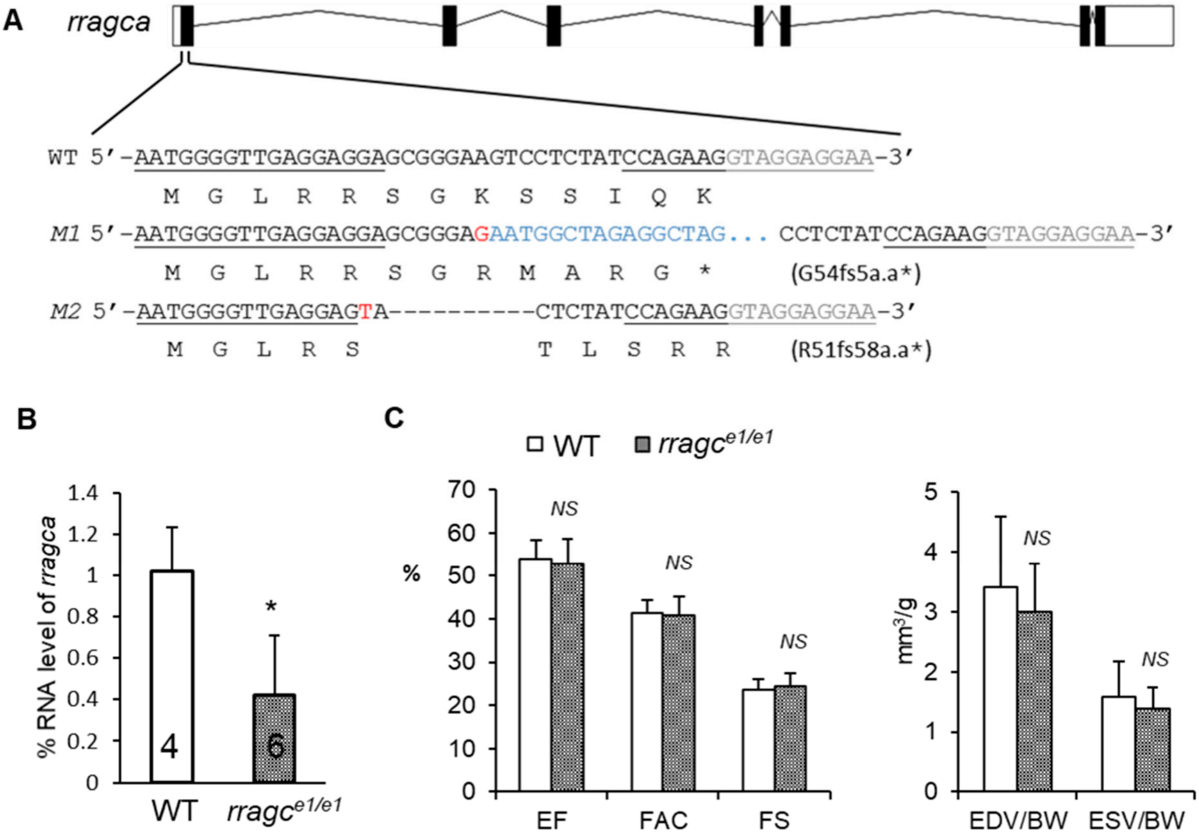

Figure S3. *rragca* knock outs do not manifest cardiac phenotypes. (**A**), Schematics of the 2 *rragca* mutant alleles generated using TALEN. The sequences in the 1st exon and intron (in grey color) that were targeted by the TALEN pairs are underlined. Dashed lines indicate deleted nucleotides; nucleotides in blue indicate insertional mutations; nucleotides in red indicate additional mutations. fs, frameshift. *, premature translational stop codon. (**B**), *rragc* transcript level is reduced in $rragc^{e1/e1}$ fish hearts. Shown are RT-qPCR analysis of the M1 allele. Data were normalized to *actb2* and expressed as fold change over WT, n=4, 6, Student's t test. (**C**), *rragca* knock-outs exhibits normal cardiac function. Shown are echocardiographic measurements for WT and $rragc^{e1/e1}$ fish at 1 year of age (n =10,11). EF, ejection function. FS, fractional shortening. EDV, end-diastolic volume. ESV, end-systolic volume. BW, body weight. All values are shown as means ± SD. *P<0.05 versus WT; *NS*, not significant versus WT by Student's t test.

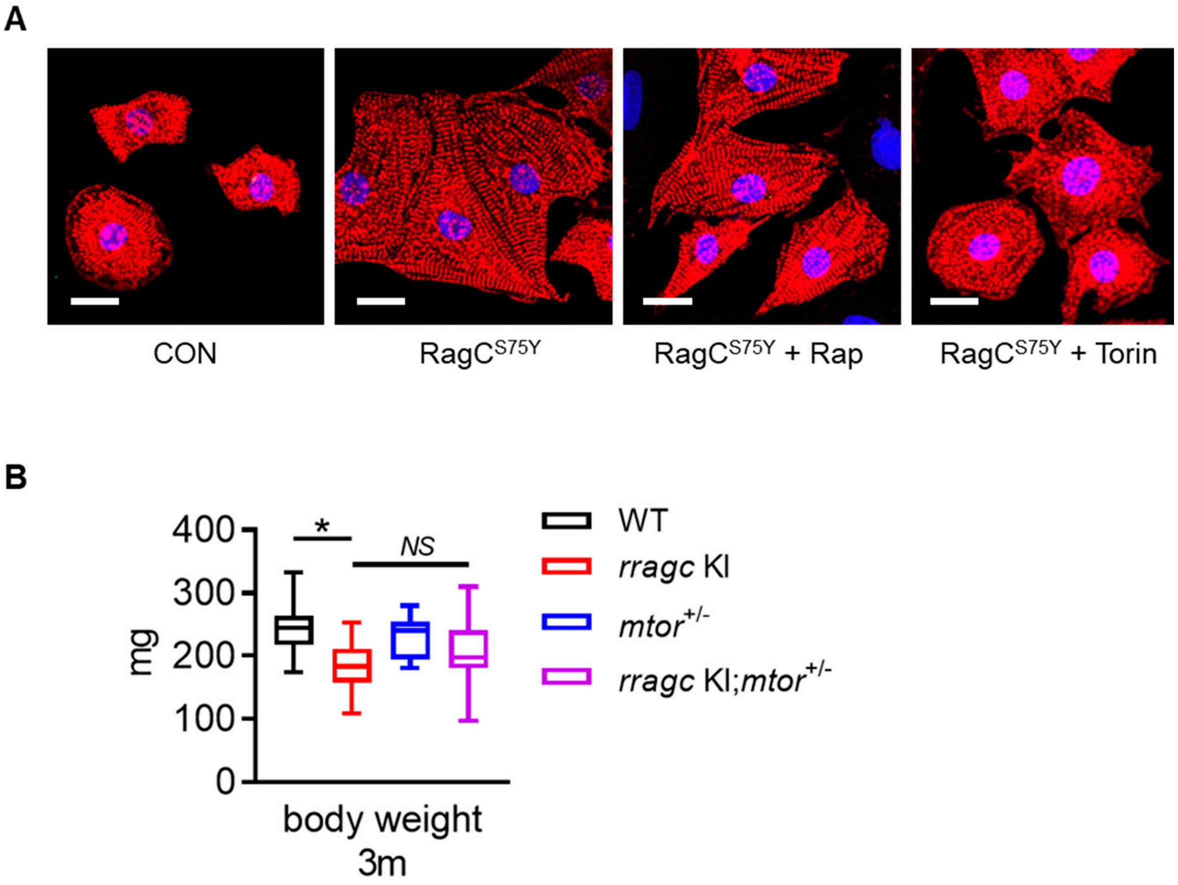

Figure S4. Effects of mTOR inhibition on phenotypes of RagC S75Y cardiomyopathy. (**A**). Representative confocal images of NRVCMs infected with recombinant adenoviruses: Ad:GFP (CON) or Ad:RagC S75Y ($RagC^{S75Y}$) for 24 hours followed by rapamycin (100 nmol/L) or torin (10 nmol/L) or vehicle incubation for another 24 hours. Cells were stained with an anti-alpha-actinin antibody (red) and DAPI (blue). Scale bar, 20 μm. (**B**). Zebrafish body weight of double mutants with their corresponding single mutants and wild type control at 3-month-old. N=19,18,9,15. Data are shown in boxplot (MIN to MAX). **P*<0.05, *NS*, not significant versus *rragc* KI, one-way ANOVA.

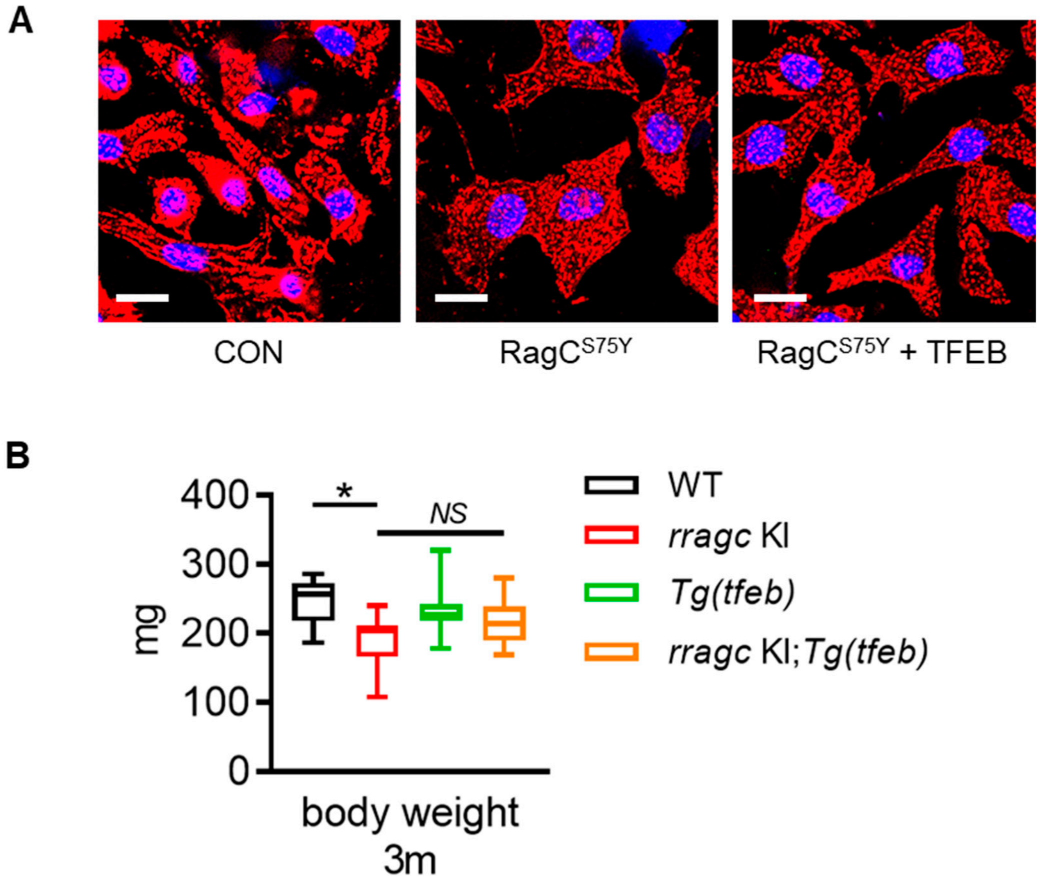

Figure S5. Effects of TFEB activation on phenotypes of RagC S75Y cardiomyopathy. (**A**). Representative confocal images of NRVCMs infected with recombinant adenoviruses: Ad:GFP (CON), Ad:RagC S75Y (RagCS75Y) for 24 hours followed by rapamycin (100 nmol/L) or torin (10 nmol/L) or Ad:TFEB-FLAG (TFEB) for 48 hours. Cells were stained with an anti-alpha-actinin antibody (red) and DAPI (blue). Scale bar, 20 µm. (**B**). Zebrafish body weight of double mutants with their corresponding single mutants and wild type control at 3-month-old. N=11,8,19,18. Data are shown in boxplot (MIN to MAX). *P<0.05, *NS*, not significant versus *rragc* KI, one-way ANOVA.

Table S1. Primers for qPCR and genotyping

Gene	Forward	Reverse
	Primers for quantitative Real Time-PCR	
Nppa	CAACACAGATCTGATGGATTTCA	CCTCATCTTCTACCGGCATC
Nppb	GTCAGTCGCTTGGGCTGT	CAGAGCTGGGGAAAGAAGAG
Mhc6	AGTGCTTCGTGCCTGATGA	AACTTGGGTGGGTTCTGCT
Mhc7	GAGGAGAGGGCGGACATT	ACTCTTCATTCAGGCCCTTG
Lamp1	TCTTCAGCGTGCAAGTCCAG	ATGAGGACGATGAGGACCAG
Lamp2a	CCAAATTGGGATCCTAACCTAA	TGGTGAAGCAGTGTTTATTAATTCC
Map1lc3b	TTTGTAAGGGCGGTTCTGAC	CAGGTAGCAGGAAGCAGAGG
Rab7a	TTACTTCGAGACCAGTGCCAAGGA	TGTCCAGTTTGATGGGTTCAGGGA
Vps18	GCTCCGCATTGACTTGGG	GCCTTCTGTCCATTGCGGT

18s	GTAACCCGTTGAACCCCATT	CCATCCAATCGGTAGTAGCG
rragca	TTGCCACTGACAGTTCACCG	GCCACTGCCGTCCTCTTTTA
nppa	GATGTACAAGCGCACACGTT	TCTGATGCCTCTTCTGTTGC
nppb	CATGGGTGTTTTAAAGTTTCTCC	CTTCAATATTTGCCGCCTTTAC
vmhc	TCAGATGGCAGAGTTTGGAG	GCTTCCTTTACAGTTACAGTCTTTC
vmhcl	GCGATGCTGAAATGTCTGTT	CAGTCACAGTCTTGCCTCCT
tfeb	TGCACCAACACACTTCCAGG	GCATGGAGAGTGCATGTTCG
lamp1	CGGTCTGTCTCCTGGCACGCATG	GTCAGAGTAATGTTGTCCTCTGTGAG
atg9a	GCAGCAGCAGAAGGGATAGTGTG	TATTTCTCCCAGCAGCATACAGG
atg9b	TCTCTTTGCCAATCGAGCCG	CGCTCAGTGTCCTTTTAGTGC
uvrag	AACTTCCTAGAGCATGGGC	AAATCCCACTTCGGATGCG
vps11	CCCAGGGAAGAAAAAAGTCC	GCCCTTTTCATACAAATACAGC
actb2	GGTATCGTGATGGACTCTGG	TCTCCTGCTCAAAGTCAAGG
gapdh	CCACCCATGGAAAGTACAAG	CTCTCTTTGCACCACCCTTA
	Primers for genotyping	
rragc	GGACTCGTTTCCCAAAGATTTC	CTCATTCACACAGCTATCTCAG
